# SuperMimic – Fitting peptide mimetics into protein structures

**DOI:** 10.1186/1471-2105-7-11

**Published:** 2006-01-10

**Authors:** Andrean Goede, Elke Michalsky, Ulrike Schmidt, Robert Preissner

**Affiliations:** 1 Berlin Center for Genome Based Bioinformatics, 3D Data Mining Group, Institute of Biochemistry, Charité, Monbijoustr.2, 10117 Berlin, Germany

## Abstract

**Background:**

Various experimental techniques yield peptides that are biologically active but have unfavourable pharmacological properties. The design of structurally similar organic compounds, i.e. peptide mimetics, is a challenging field in medicinal chemistry.

**Results:**

SuperMimic identifies compounds that mimic parts of a protein, or positions in proteins that are suitable for inserting mimetics. The application provides libraries that contain peptidomimetic building blocks on the one hand and protein structures on the other. The search for promising peptidomimetic linkers for a given peptide is based on the superposition of the peptide with several conformers of the mimetic. New synthetic elements or proteins can be imported and used for searching.

**Conclusion:**

We present a graphical user interface for finding peptide mimetics that can be inserted into a protein or for fitting small molecules into a protein. Using SuperMimic, promising locations in proteins for the insertion of mimetics can be found quickly and conveniently.

## Background

Many protein interactions are known, mostly involving other proteins, peptides or different organic molecules, and more and more are being deciphered. The main goal of drug design is to interfere specifically with these interactions. As peptides are often poor drug candidates, the need arises for bioequivalent compounds with better pharmacological properties. Starting from a known spatial structure, the aim is to find compounds that mimic the function of a peptide but have improved cellular transport properties, low toxicity, few side effects and more rigid structures as well as protease resistance [1,2].

Various methods exist for developing peptide mimetics. These include computational as well as experimental screening methods. One method is to identify small peptides that are essential for the interactions of the protein, e.g. using SPOT synthesis. Subsequently, mimetics for these peptides are designed that can be used as drugs. On the basis of a known protein structure, scaffolding templates for binders can also be constructed and then optimised using different methods (see [3-5] for reviews).

The approach presented in this paper is to detect peptide mimetics directly using a known protein structure and a mimetic structure. Specific atomic positions are defined in both structures and then compared with respect to their spatial conformations. In this way, organic compounds that fit into the backbone of a protein can be identified. Conversely, it is possible to find protein positions where a specific mimetic could be inserted.

A practical application of SuperMimic could be the design of an artificial protein in which peptidomimetic building blocks replace parts of the backbone and that can subsequently be synthesized. Moreover, it is possible to find organic compounds or design artificial peptides that imitate the binding site and hence the functionality of a protein.

A library containing peptidomimetic building blocks collected from the literature and represented by several conformations, as well as several protein structural libraries, are made available. Both libraries can be scanned exhaustively. The searches can also be performed with structures provided by the user.

## Implementation

### Protein and mimetic libraries

Using the program SuperMimic, collections of short chains of PDB structures [6] as well as peptide mimetics can be scanned. In order to guarantee rapid access to 3D data, all libraries are stored in binary form. In addition, the address of each protein chain within the binary file is stored and imported together with a list of the chains at the start of the program. Thus, samples of proteins from the library can be scanned at low expense.

Peptide mimetic structures are arranged in sub-libraries saved in separate files and automatically loaded after the program is started. This facilitates regular fast updates of the libraries by creating new files.

### Program

**Figure 1:**
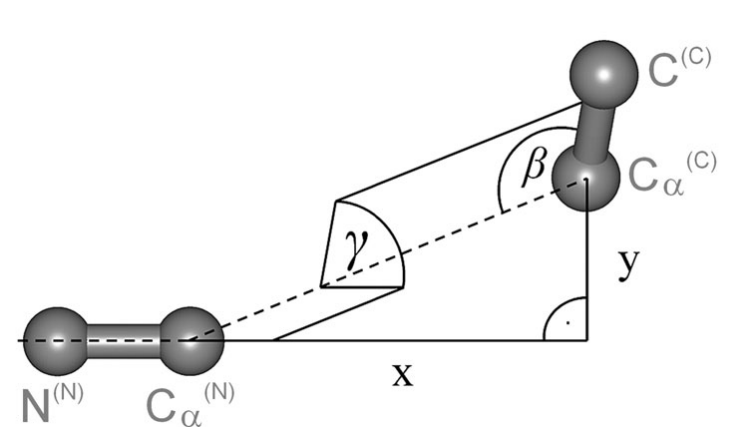
**Geometric values that are evaluated and compared during the primary search. **Atoms N^(N) ^and C_α_^(N) ^are part of the first replaced amino acid; C_α_^(C) ^and C^(C) ^are part of the last replaced amino acid on the protein side and are the corresponding atoms on the mimetic side. The *x-y *plane of the coordinate system is defined by the points N^(N)^, C_α_^(N) ^and C_α_^(C)^, where the *x*-axis connects N^(N) ^and C_α_^(N)^. The main characteristic values are the distances *x *and *y*. Further characteristic values are β, the angle included by the lines connecting the atoms C_α_^(N) ^and C_α_^(C) ^and also C_α_^(C) ^and C^(C)^, and γ, the dihedral angle between the N^(N) ^- C_α_^(N) ^- C_α_^(C) ^and C_α_^(N) ^- C_α_^(C) ^-C^(C) ^planes.

Screening is based on spatial superposition of four so-called stem atoms of the proteins with the analogous atoms of the peptide mimetics. In the case described here, the stem atoms are the N and C_α _atoms of the first amino acid to be mimicked and the C_α _and C atoms of the last. The stem positions are represented by four parameters: two distances, *x *and *y*, and two angles, β and γ, as shown in Figure 1. These parameters are computed rapidly for all positions within the protein, and for all conformations of all chosen mimetics.

The 'goodness' of a pair of stem positions is then evaluated on the basis of these parameters by the formula

*goodness *= Δ*x*^2 ^+ Δ*y*^2 ^+ 2(Δβ^2 ^+ Δγ^2^),

where e.g. Δ*x*^2 ^denotes the squared deviation of the *x *values. The square root of the goodness is an upper estimate of the Root Mean Square Deviation (RMSD) of the stem atoms. A detailed description of the procedure can be found in [7].

In this way, a pre-selection of suitable candidates is obtained. This primary search permits rapid calculations because the evaluation of goodness is significantly less expensive than that of RMSD. Pairs of stem atoms yielding a goodness below a given limit are retained and their RMSD is calculated according to the algorithm described by Kabsch [8]. These calculations can also be performed very rapidly, as the required spatial coordinates are stored in the main memory.

The procedure described so far is carried out for each chosen protein or protein chain, and the hits are collected. Finally, they are reordered according to the RMSD of the stem atoms. Different goodness limits in the primary search are set depending on the kind of the search, so that the set of hits is restricted to a reasonable size.

## Results and Discussion

### Peptide mimetics libraries

SuperMimic provides a library of 126 peptidomimetic structures. It contains 88 synthetic elements described in the literature, which have been arranged in sublibraries such as beta-turn- or gamma-turn-mimetics. Some of them are known to be drug-like compounds. Appropriate references can be found on the website. Moreover, the library contains a collection of 18 peptides, each comprising a sequence of one D-amino acid flanked by two L-amino acids, which can be used as beta- or gamma-turn mimetics, and 20 peptidomimetic ligands extracted from PDB structures. In order to account for the flexibilities of the peptide mimetics, each structure contained in the library is represented by 5–13 low-energy conformers. These were generated by the Accelrys software MedChem Explorer, using the algorithm of Smellie et al. [9].

### Protein libraries

Insertion of peptide mimetics into proteins is realised by chemical syntheses. Such syntheses are mostly practised with small proteins, so it is useful to restrict a search to small protein chains. To allow candidates for synthesis to be identified easily and rapidly, the program is linked to a library of such proteins. This library contains 10403 chains of PDB structures [6] up to 100 amino acids long. Alternatively, this large library can be replaced by a set of 2206 chains with less than 90% sequence identity, represented by structures with best resolution, or by a set of 416 chains with less than 30% sequence identity. All protein chain sets were generated using the Columba database [10].

### Searching options

SuperMimic permits two general searching approaches. Firstly, it is possible to conduct a fast scan for small molecules that mimic the structure of a given peptide or can be inserted into a given protein or peptide. Secondly, starting with a peptidomimetic structure, positions in proteins suitable for its insertion can be screened. There are several options for the screening process.

Forward searches:

1. A protein structure can be imported by the user, either from the libraries of small proteins provided or by loading a PDB file. A search for peptide mimetics that fit into the backbone of the chosen protein can then be initiated. This results in a list of peptide mimetics, the position within the protein where the mimetic could be inserted, and the conformation of the mimetic that fits best.

2. Instead of scanning the whole protein structure the search can be limited to a special part of the protein, e.g. an exposed loop.

3. The stem positions within the protein can be fixed. In this case the position is not limited to the backbone. Arbitrary atoms can be chosen as stem atoms, including those in the protein side chains. This option can be used if the position within the protein where a mimetic structure should be fitted is known exactly.

4. All the above-described searches can be performed within the whole mimetics library or alternatively limited to a sublibrary of mimetics, e.g. beta-turn-mimetics, or even to an individual molecule.

Backward searches:

5. The structure of a mimetic can be imported by the user, either from the libraries of peptide mimetics provided or by loading the structure of a small molecule in MDL mol or sd file format. A search for proteins where the mimetic fits into the backbone can then be initiated. This results in a list of proteins, including the position within each protein where the mimetic could be inserted, and the conformation of the mimetic that fits best.

6. Instead of the whole library of small protein chains the search can be limited to a sample of proteins from the library, or to an individual protein.

All-to-all searches:

7. All-to-all comparisons are also possible, but owing to the large number of hits this can be limited by the memory capacity of the computer. Should this situation arise, such comparisons may be restricted to samples from the protein library on one side, or to sub-families of peptide mimetics on the other.

Stem atoms have been predefined for all the libraries provided and should be specified interactively by the user for his or her own structures. Delivering several conformers will yield better results as the search space is enlarged.

All possible combinations of protein and mimetic stem atoms are scanned and candidates fulfilling certain geometrical criteria are sorted according to the Root Mean Square Deviation (RMSD) of the stem atoms. They can be inspected visually in a graphical display. Possible clashes between atoms of the mimetic and the protein are indicated. The superposed proteins and mimetics can be exported as complexes in PDB file format; alternatively, the mimetics can be saved as MDL mol files with their atoms in the protein's coordinate system.

### Supporting website

Two versions of the program can be downloaded from the SuperMimic website. With the standard version, fragments of 2–6 amino acid residues can be replaced with peptide mimetics. The extended version handles peptides up to twelve residues long. By bridging larger sequences, the search space is enlarged at the expense of computing time.

Furthermore, all the protein and peptide mimetics libraries are available on the website. Different mimetics sublibraries can be included or excluded by retaining or omitting the respective files. Library files only have to be saved in the same directory as the executable file. They are loaded automatically at subsequent program starts.

In addition, descriptions of the peptide mimetics can be found on the website, including structures, names, classifications described in the literature and references. For support, help pages and several demonstrations explaining how to use the program are provided.

### Performance

A typical search for the insertion positions of one peptide mimetic structure in the large protein library comprises a comparison of roughly 10000 protein chains, each less than 100 amino acids long, with an average of ten conformers of the mimetic. With the standard version of SuperMimic, peptides of 2–6 amino acids can be bridged, resulting in nearly 500 possible stem positions in one protein chain. Thus, 50 million geometrical comparisons are necessary. Owing to the effective ways of storing the data and pre-selecting the fitting stem positions used in SuperMimic, such a search only takes about three minutes on a low-end desktop PC (Athlon 1400).

Limiting the similarity search to four stem positions allows the screening of large sets of structures in a short time. This is possible because the positions of these four atoms can be described and compared easily using only six parameters, two of which are fixed bond lengths [7].

## Conclusion

SuperMimic is a tool for finding potential non-peptidic building blocks that can replace or mimic parts of a protein, and conversely for identifying locations within a protein where such building blocks can be inserted. It allows rapid, convenient searches within the protein and peptide mimetic libraries provided, as well as using imported structures.

## Availability and requirements

**Project name: **SuperMimic

**Project home page: **

**Operating system(s): **Windows

**Programming language: **Delphi

**Other requirements: **no

**License: **no

**Any restrictions to use by non-academics: **no

## Authors' contributions

AG created the database structures and the program, web site and demonstrations and helped to draft the manuscript. EM drafted the manuscript and helped to create the web site and the library of peptide mimetics. US created the library of peptide mimetics and collected the literature references for the mimetics. RP conceived of the project, participated in its design and coordination and helped to draft the manuscript. All authors read and approved the final manuscript.

## Figures and Tables

**Figure 2:**
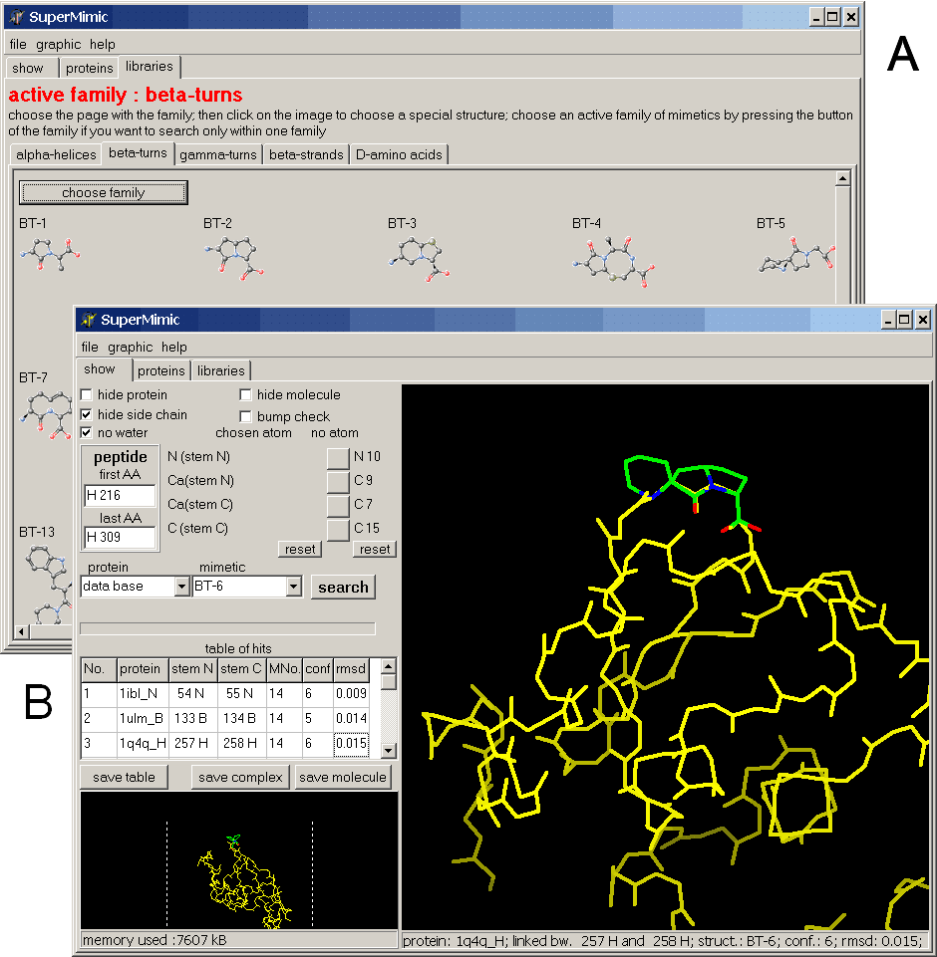
**Screenshots of the SuperMimic program. A. **Screenshot of the libraries of peptide mimetics, showing the family of beta-turn-mimetics. Searches can be performed within all conformations of one structure, within a family of mimetics, or within the whole library of mimetics. **B. **Screenshot after a search for suitable positions of a beta-turn-mimetic within the internal library of about 10403 PDB chains. The picture shows an apoptosis 1 inhibitor from Drosophila melanogaster, PDB-code 1q4q, chain H (yellow), with amino acids 257 and 258 replaced by the mimetic (green).
